# Supplementary figures and images for: The central pore of HIV-1 capsomers promotes sustained stability of the viral capsid
Source: bioRxiv. 2025 May 19:2025.05.19.654868. Preprint. [Version 1] doi: 10.1101/2025.05.19.654868 (PMC12139970; doi:10.1101/2025.05.19.654868)

Figure S1

**A**

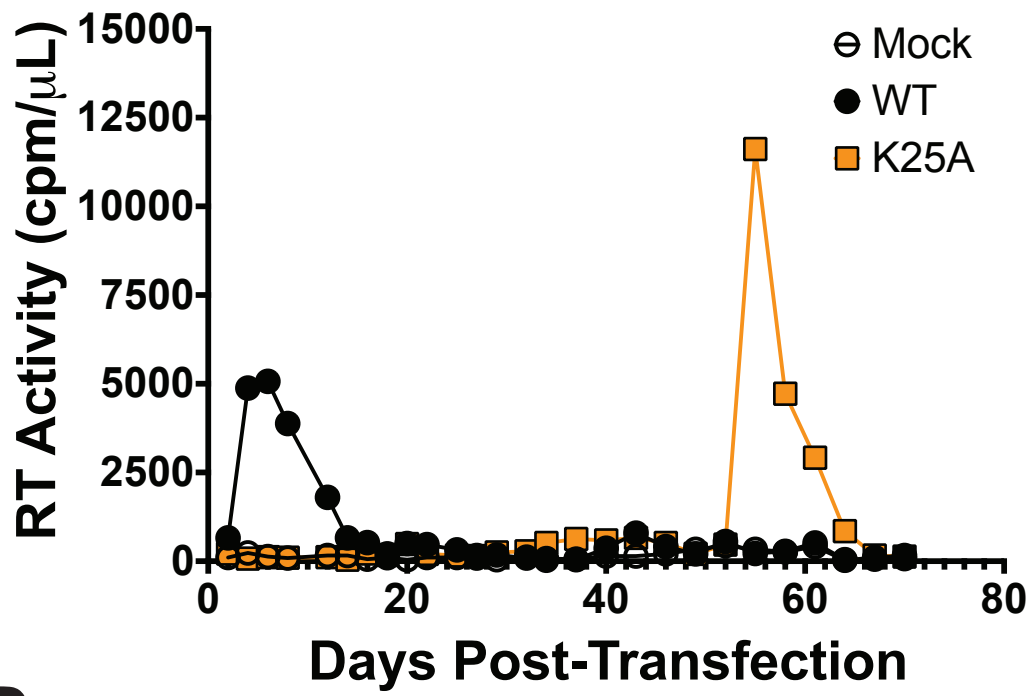

**B**

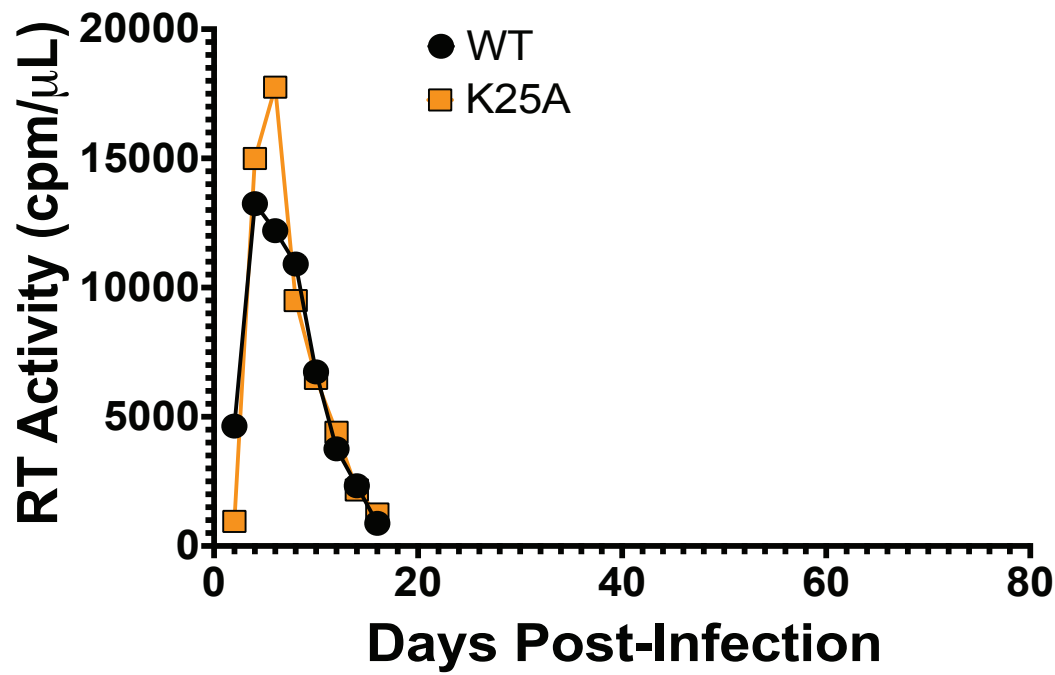

Supplement: Supplement 1 — Figure S1. K25A acquires N21K upon propagation in MT4 cells. (A) MT4 cells were transfected with WT or CA-K25A infectious molecular clones (NL4-3) and viral replication was monitored by RT assay. (B) WT or K25A virus collected from the peak of replication in (A) were used to infect MT4 cells and viral replication was monitored by RT assay. [file media-1.pdf]

Figure S2

A

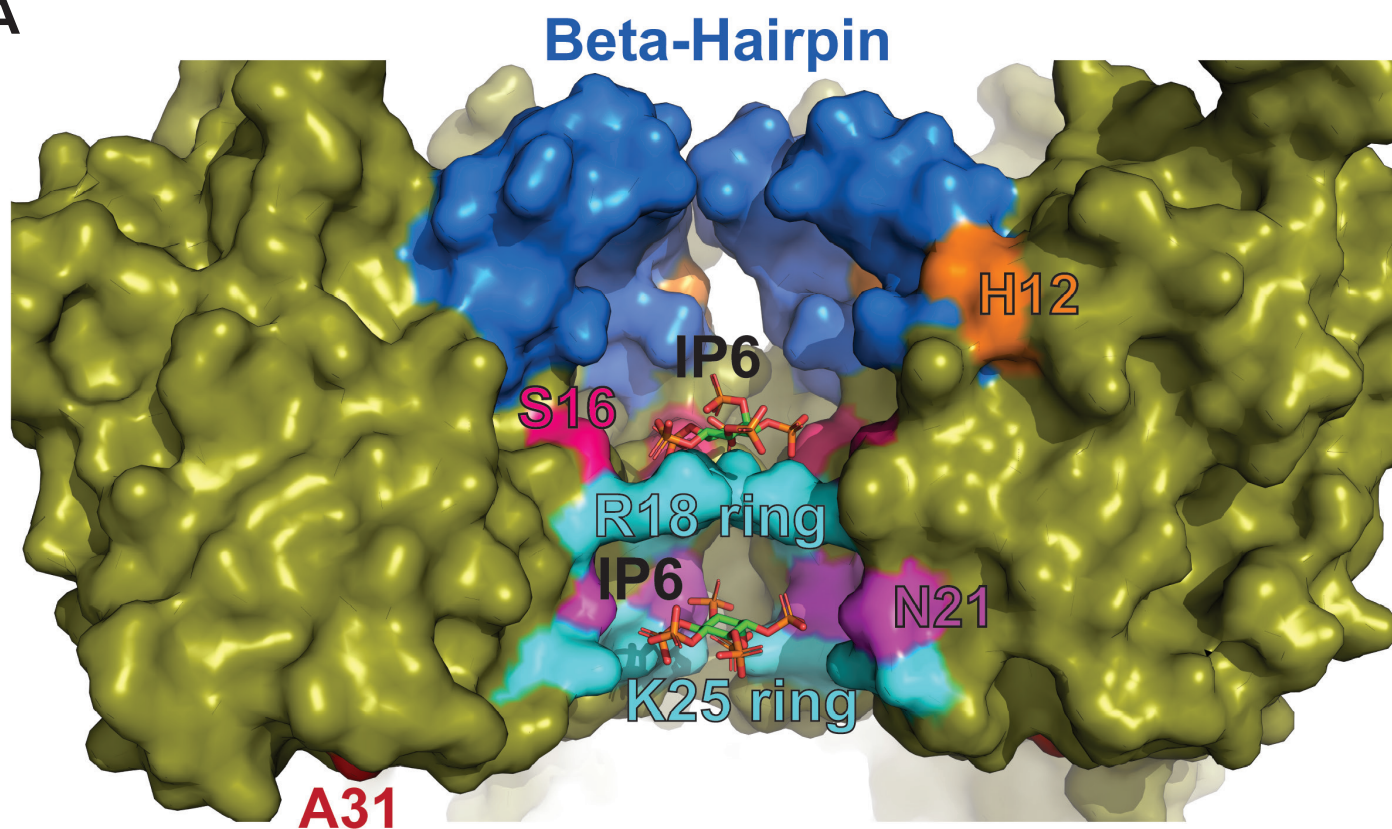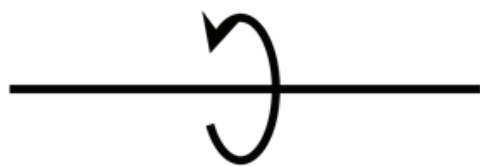

B

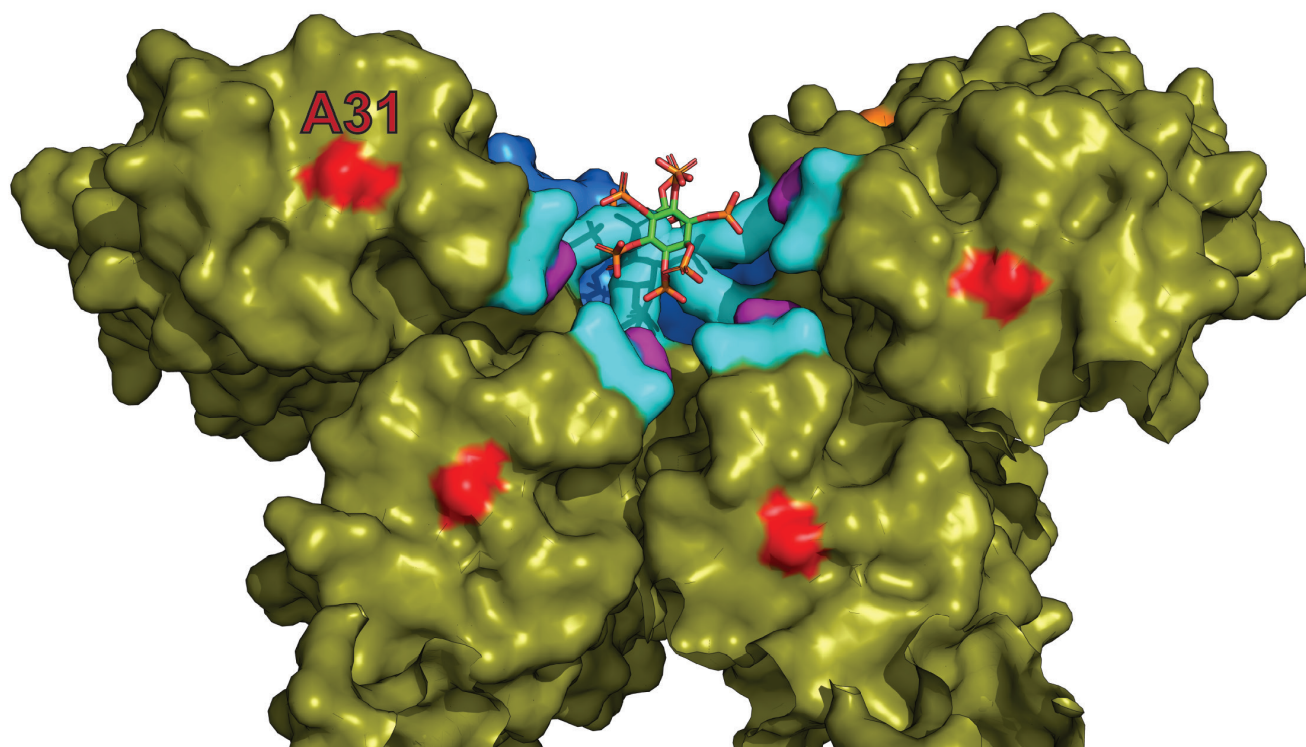

Supplement: Supplement 3 — Figure S2. Locations of compensatory mutations acquired upon propagation of R18A/N21K and R18S/N21K. (A) Cross section of the central pore of the CA hexamer (based on PDB 6R6Q) showing the locations of H12 (orange), S16 (pink), and N21 (magenta) relative to the R18 and K25 IP6-binding rings. (B) 90° rotation of (A) to depict the location of A31T (red) on the luminal surface of the CA hexamer. [file media-3.pdf]

Figure S3

A

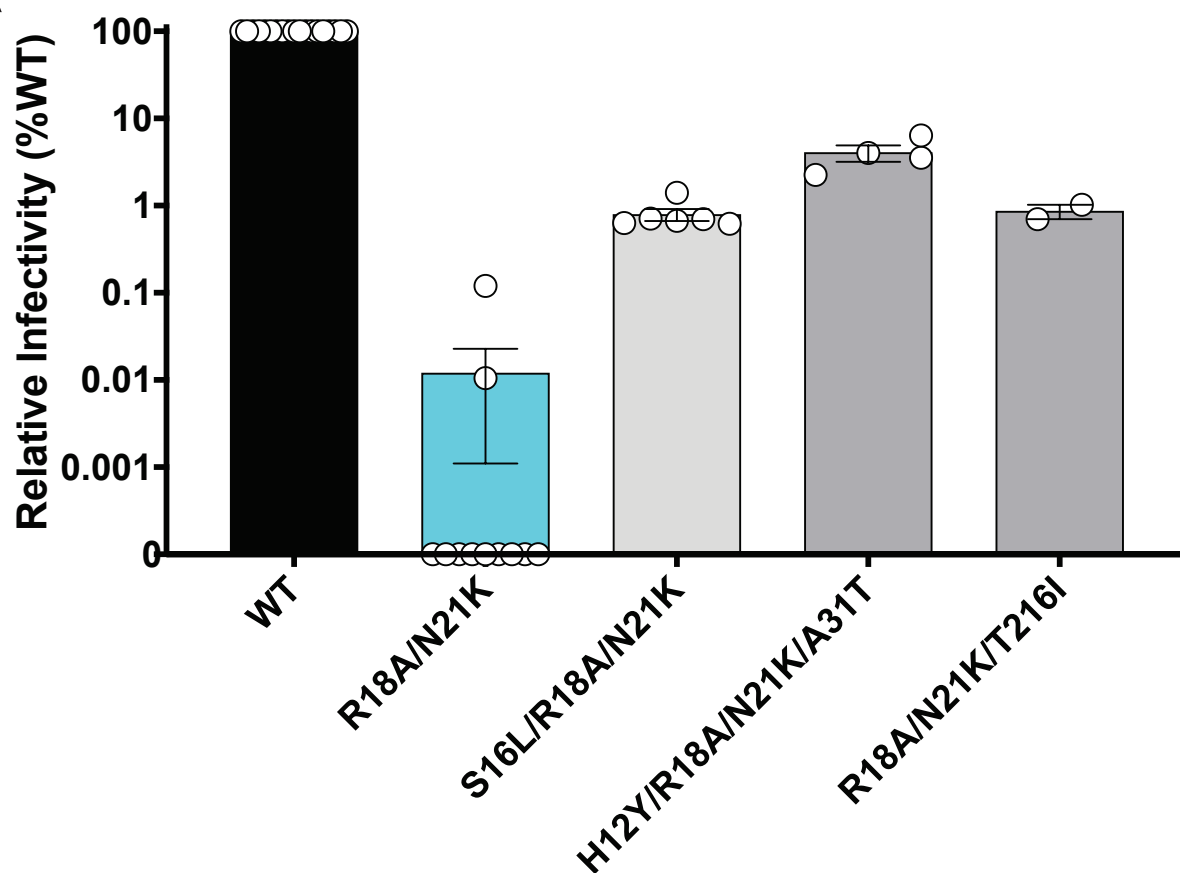

B

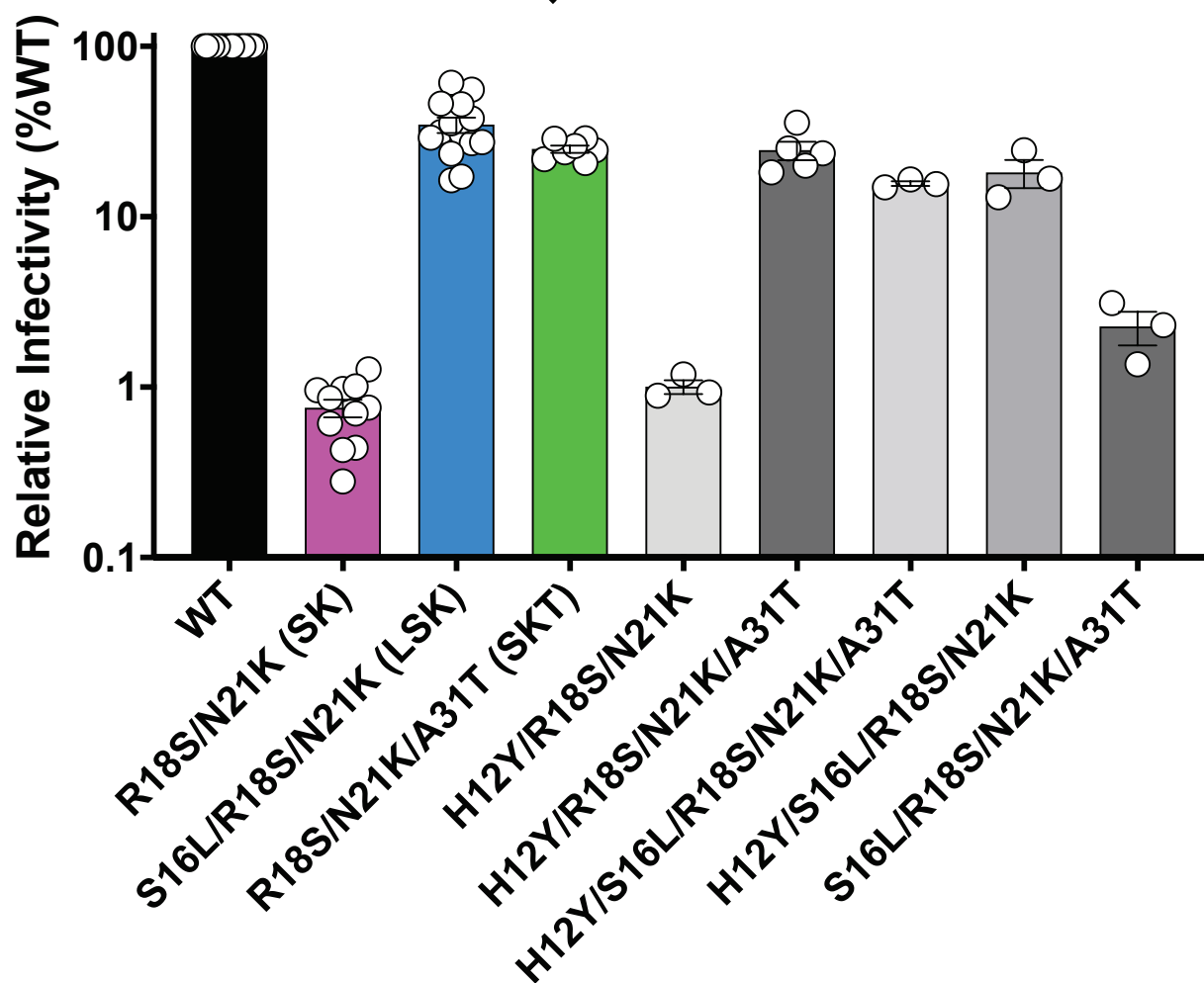

Supplement: Supplement 4 — Figure S3. A serine at position 18 facilitates enhanced rescue by compensatory mutations. Single-cycle infectivity of HIV-1 CA mutants harboring the R18A/N21K changes (A) or the R18S/N21K (B) changes with compensatory mutations identified in this study quantified by luminescence in infected TZM-bl cells. Virus stocks were normalized by RT assay prior to infection of TZM-bl cells. The R18A/N21K data presented for comparison in (A) are the same that is presented in Figure 1B. The SK, LSK, and SKT data presented for comparison in (B) are the same that is presented in Figure 1H. Error bars depict the mean ± s.e.m. from at least 3 independent measurements. Abbreviations: R18S/N21K (SK); S16L/R18S/N21K (LSK); R18S/N21K/A31T (SKT). [file media-4.pdf]

# Figure S4

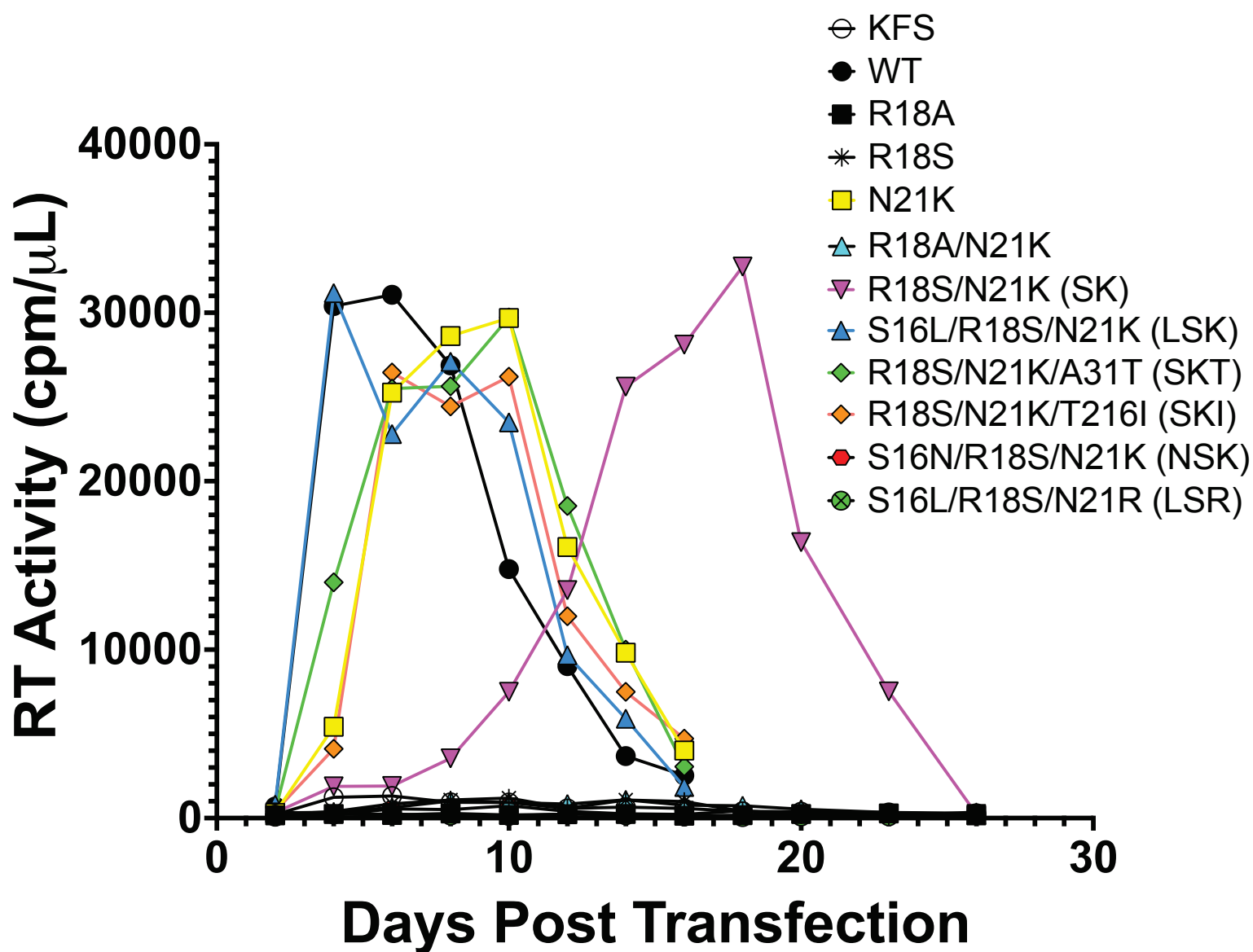

Supplement: Supplement 5 — Figure S4. Compensatory mutations restore replication in MT4 cells. Representative spreading infection experiment depicting the replication kinetics of WT HIV-1 or defective (R18A, R18S, R18A/N21K, SK, NSK, LSR) and rescued (LSK, SKT, SKI) central pore mutants in MT4 cells. Spreading infection experiments were initiated by transfection of MT4 cells with infectious molecular clones and replication kinetics were monitored by RT assay. The Env(−) clone pNL4-3/KFS is included as a negative control. Abbreviations: R18S/N21K (SK); S16L/R18S/N21K (LSK); R18S/N21K/A31T (SKT); R18S/N21K/T216I (SKI); S16N/R18S/N21K (NSK); S16L/R18S/N21R (LSR). [file media-5.pdf]

# Figure S5

## Donor 1

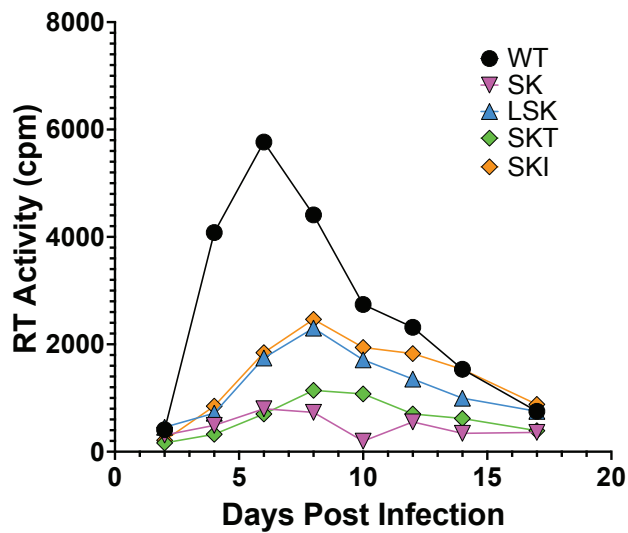

## Donor 2

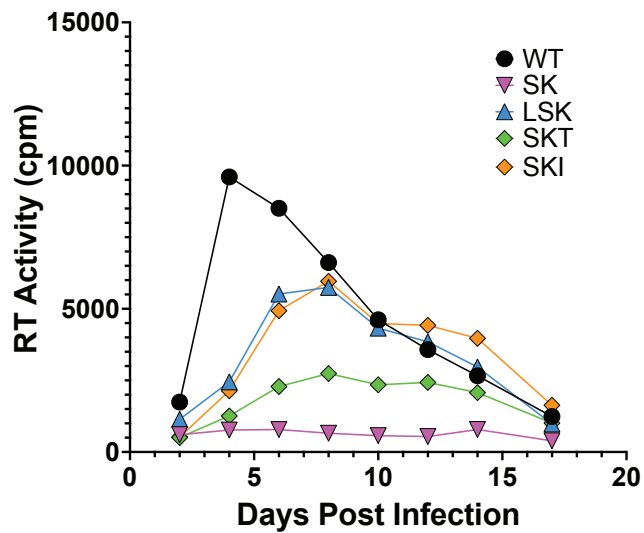

## Donor 3

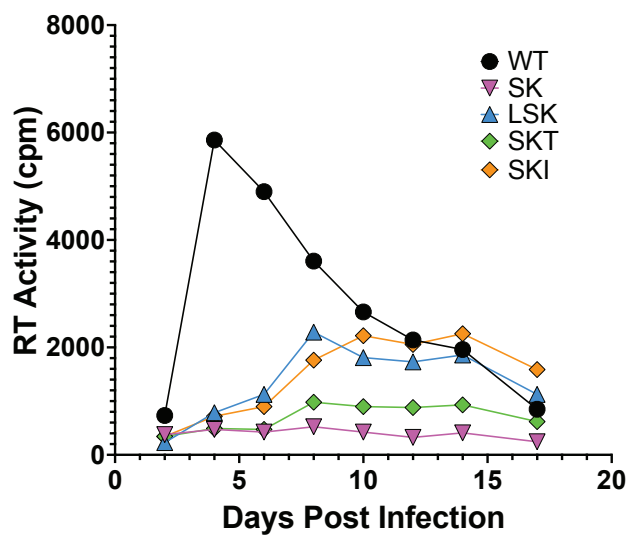

## Donor 4

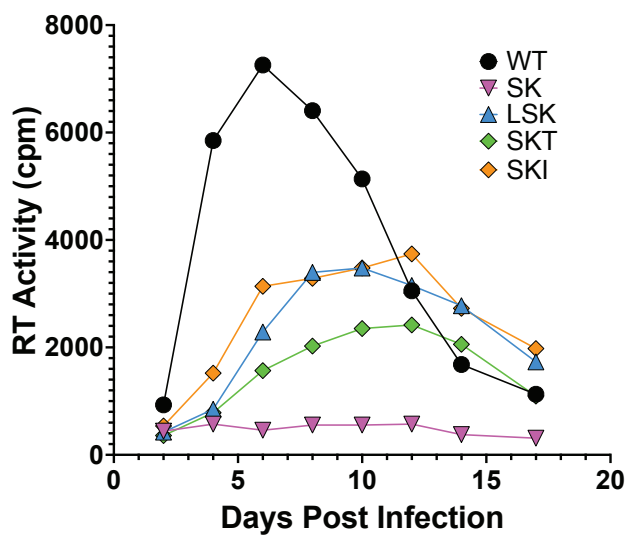

Supplement: Supplement 6 — Figure S5. Compensatory mutations partially restore replication to R18S/N21K (SK) in primary peripheral blood mononuclear cells (PBMCs). Cells isolated from whole blood collected from 4 individual donors were activated for 5 days with PHA-P and infected with RT-normalized WT, SK, LSK, SKT, or SKI. Replication kinetics were monitored by RT assay. Abbreviations: R18S/N21K (SK); S16L/R18S/N21K (LSK); R18S/N21K/A31T (SKT); R18S/N21K/T216I (SKI). [file media-6.pdf]

Figure S6

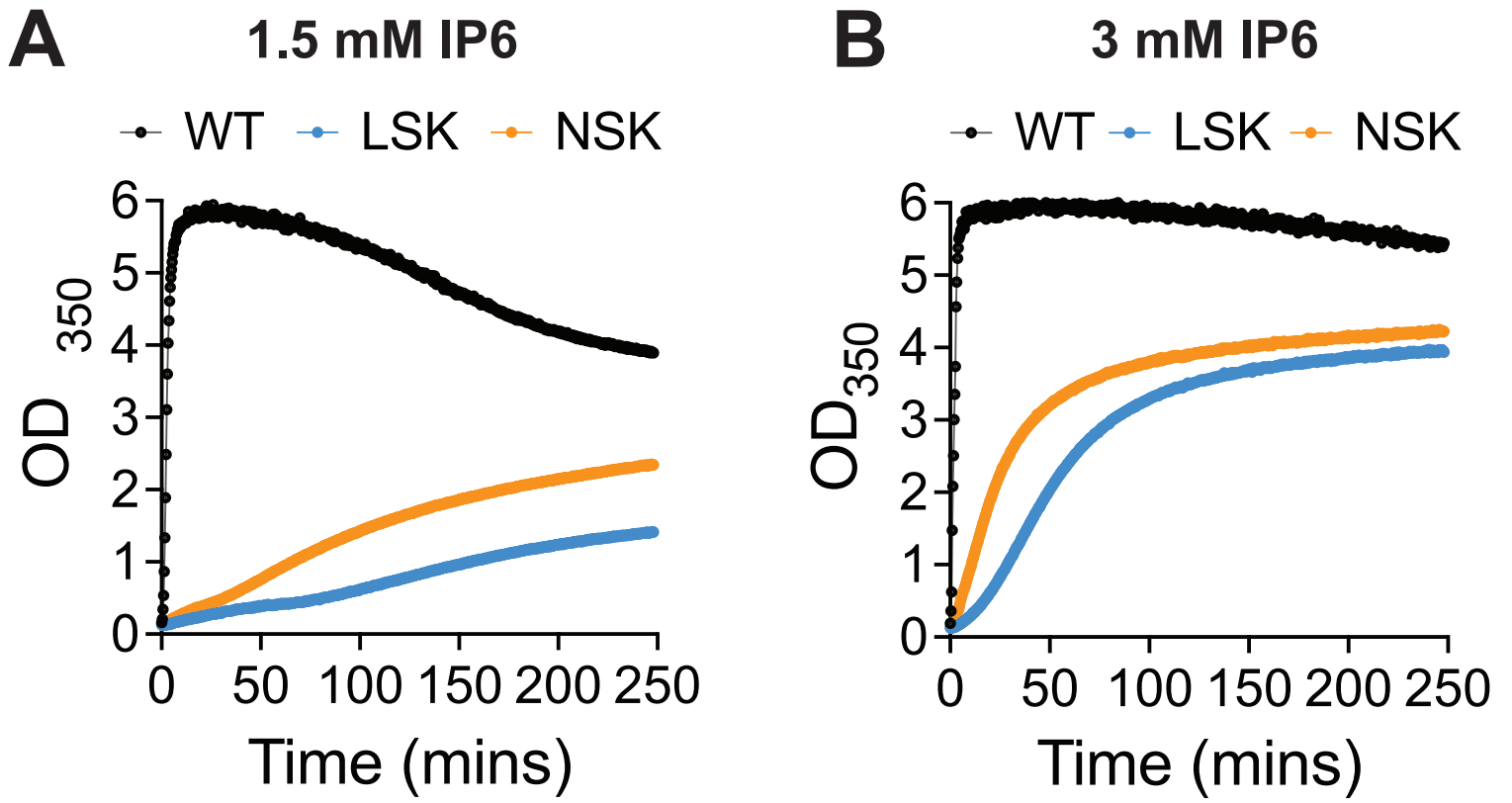

Supplement: Supplement 7 — Figure S6. LSK and NSK CA assembles less efficiently than WT CA at low IP6 concentrations. In vitro assembly kinetics of 100 μM of the indicated recombinant mature CA protein in the presence of 1.5 mM (A) and 3 mM (B) IP6, as determined by measuring absorbance at 350 nm over time. Abbreviations: S16L/R18S/N21K (LSK); S16N/R18S/N21K (NSK). [file media-7.pdf]

Figure S7

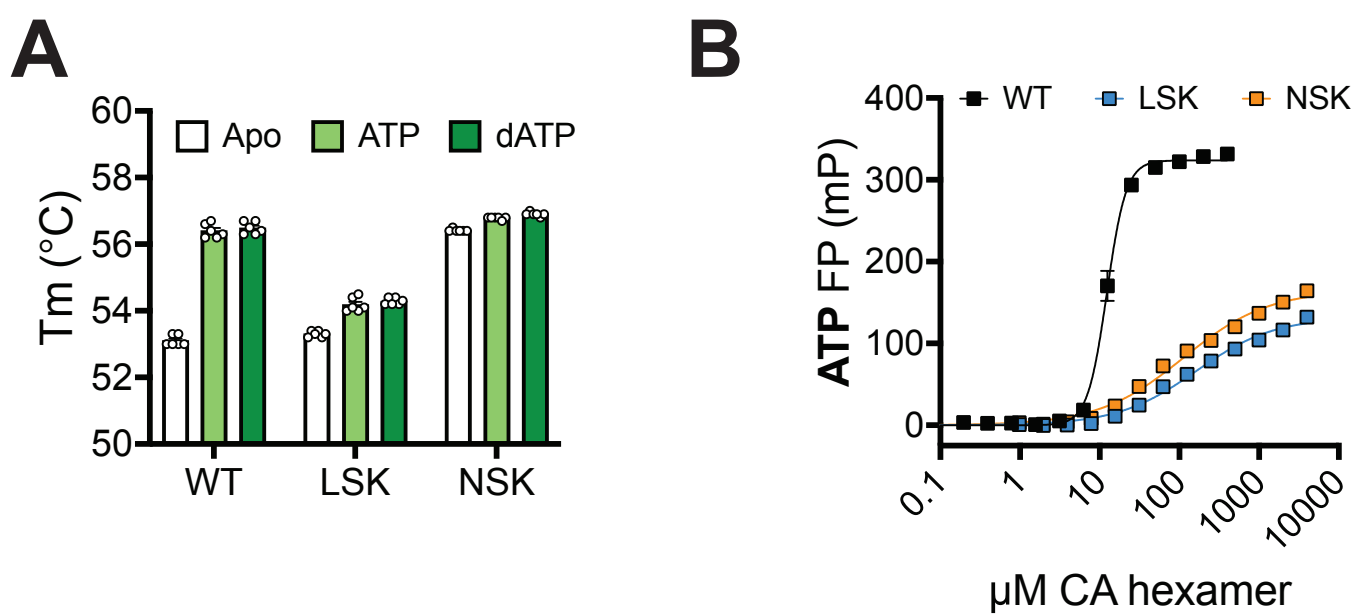

Supplement: Supplement 8 — Figure S7. LSK and NSK crosslinked CA hexamers bind weakly to ATP and dATP. (A) Thermal stability measurements for the indicated crosslinked CA hexamers alone or in the presence of ATP or dATP, as measured by differential scanning fluorimetry. Error bars depict the mean ± s.e.m. from at least 3 independent experiments. (B) ATP binding kinetics for the indicated crosslinked CA hexamers, as measured by fluorescence polarization. Error bars depict the mean ± s.e.m. from at least 3 independent experiments. Abbreviations: S16L/R18S/N21K (LSK); S16N/R18S/N21K (NSK). [file media-8.pdf]

Figure S8

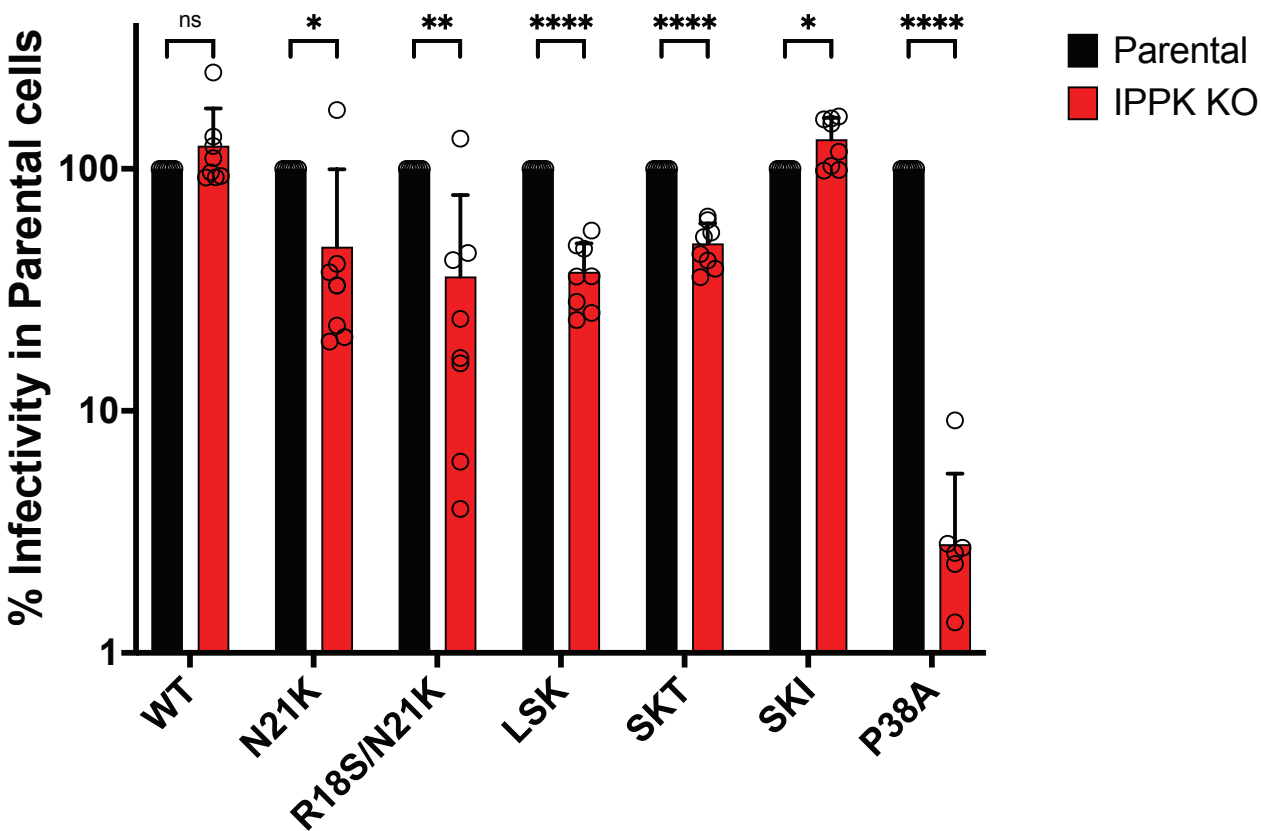

Supplement: Supplement 9 — Figure S8. Rescued central pore mutants display mild IP6 dependency in target cells. VSV-G pseudotyped, luciferase-encoding virions were used to infect 293T parental and IPPK KO target cells. Specific infectivity was measured at 48 hours post-infection by quantifying luminescence after lysis of infected cells. Infectivity for each virus in IPPK KO target cells is expressed as a percentage of its infectivity in parental cells. Error bars depict the mean ± s.e.m. from 8 independent experiments. Statistical significance was determined by unpaired Student’s t-test (p-value summary: >0.05 = not significant; <0.05 = *; <0.01 = **; <0.001 = ***; <0.0001 = ****. Abbreviations: R18S/N21K (SK); S16L/R18S/N21K (LSK); R18S/N21K/A31T (SKT); R18S/N21K/T216I (SKI). [file media-9.pdf]
