# Supplementary material for: The central pore of HIV-1 capsomers promotes sustained stability of the viral capsid
Source: bioRxiv. 2025 May 19:2025.05.19.654868. Preprint. [Version 1] doi: 10.1101/2025.05.19.654868 (PMC12139970; doi:10.1101/2025.05.19.654868)
Supplement: Supplement 2 — Table S1. CA mutations acquired by central pore mutants subjected to forced evolution. MT4 cells were transfected with infectious molecular clones harboring the indicated mutations. Virus collected from the peak of replication was used to infect fresh MT4 cells, and the process was repeated until replication kinetics resembled WT. Compensatory mutations acquired upon propagation were identified by Sanger sequencing of GagPol DNA fragments amplified by PCR from genomic DNA isolated from cells at the peak of viral replication. HIV-1 mutant clones used to initiate each forced evolution experiment in this study are listed along with the number of passages and compensatory mutations identified in CA. [file media-2.pdf]

Table S1

Transfected Clone	Passages	CA Mutations Acquired
K25A	2	N21K
R18A/N21K	3	S16L, A18S
R18A/N21K	3	H12Y, A31T
R18A/N21K	3	L56V, E187K
R18S/N21K	2	T216I
R18S/N21K	2	T216I
R18S/N21K	2	A31T
R18S/N21K	2	I15M
S16N/R18S/N21K	2	N16L
S16L/R18S/N21R	2	R21K
